# Supplementary material for: Virulence strategies of an insect herbivore and oomycete plant pathogen converge on host E3 SUMO ligase SIZ1
Source: New Phytol. 2022 May 28;235(4):1599–614. doi: 10.1111/nph.18184 (PMC9545238; doi:10.1111/nph.18184)
Supplement: Supplementary file 1 — Fig. S1 Amino acid alignment of different versions of SIZ1 and fragments recovered from yeast‐two‐hybrid screens. Fig. S2 Confirmation of interactions between aphid effector Mp64 and AtSIZ1 (Arabidopsis thaliana) or NbSIZ1 (Nicotiana benthamiana) in yeast through activation of various reporter genes. Fig. S3 Virus‐induced gene silencing of NbSIZ1 is effective and reduces host susceptibility to Phytophthora capsici. Fig. S4 Representative images of Arabidopsis mutant lines infected with Phytophthora capsici. Fig. S5 Amino acid alignment of Mp64 and predicted orthologues in aphid species. Fig. S6 Reverse transcription‐polymerase chain reaction (RT‐PCR) confirms expression of aphid effector Mp64 in transgenic Arabidopsis lines and plant phenotypes. Fig. S7 Expression of CRN83_152 but not Mp64 in Nicotiana benthamiana enhances host susceptibility to Phytophthora capsici. Fig. S8 Localization of effectors Mp64 and CRN83_152(6D10) and AtSIZ1 in the host nucleus. Fig. S9 Expression of full length SIZ1‐RFP fusion proteins in planta. Fig. S10 Mp64 but not CRN83‐152_6D10 enhanced SIZ1 stability in planta. Fig. S11 CRN83_152_6D10 enhances AtSIZ1 triggered cell death in Nicotiana benthamiana. Fig. S12 CRN83_152_6D10 enhances SIZ1‐mediated SUMOylation Table S1 Primers used in this study. Table S2 Plasmid constructs used in this study. Table S3 Candidate interactors of Mp64 and CRN83_152 identified in yeast‐two‐hybrid screens against a Nicotiana benthamiana prey library. Please note: Wiley Blackwell are not responsible for the content or functionality of any Supporting Information supplied by the authors. Any queries (other than missing material) should be directed to the New Phytologist Central Office. [file NPH-235-1599-s001.pdf]

New Phytologist Supporting Information

Virulence strategies of an insect herbivore and oomycete plant pathogen converge on host E3 SUMO ligase SIZ1

Shan Liu, Camille J.G. Lenoir, Tiago M.M.M Amaro, Patricia A. Rodriguez, Edgar Huitema, Jorunn I.B. Bos

Article acceptance date: 15 April 2022

Table S1. Primers used in this study.

Primer name	Sequence
DONR-Mp64_F	5'-CAAATGTTCTCAATTTTTTCCGAAAAT-3'
DONR-Mp64_Rev	5'-GGGGACCACTTTGTACAAGAAAGCTGGGTCTTAATAATAGCGTCGTCTTTG-3'
NbSIZ1-attB1	5'-AAAAAGCAGGCTTCACCATGGATTTGGTTGCTAGT-3'
NbSIZ1-attB2	5'-AGAAAGCTGGGTCCTATTCAGAATCCGAATCAATA-3'
NbSIZ1-attB2-no stop	5'-AGAAAGCTGGGTCTTCAGAATCCGAATCAATACTC-3'
attB1	5'-GGGGACAAGTTTGTACAAAAAAGCAGGCT-3'
attB2	5'-GGGGACCACTTTGTACAAGAAAGCTGGGT-3'
pDONR207-F	5'-TCGCGTTAACGCTAGCATGGATCTC-3'
pDONR207-R	5'-TGTAACATCAGAGATTTTGAGACAC-3'
pLexA-N-F	5'-CTTCACCATTGAAGGGCTGG-3'
pLexA-N-R	5'-AAATCATAAGAAATTCGCC-3'
pGAD-HA-F2	5'-GATCTTTAATACGACTCACTATAG-3'
pGAD-HA-R2	5'-TCGATGGATCCCGTATCGATG-3'
pGWB-F	5'-CATTTGGAGAGAACACGG-3'
p35s-F	5'-TTCGCAAGACCCTTCCTCTA-3'
T35s-R	5'-ACTGGTGATTTTTGCGGACT-3'
GFP-Nter-F	5'-ACGTAAACGGCCACAAGTTC-3'
GFP-Cter-a-R	5'-GAACTTGTGGCCGTTTACGT-3'
RFP-RevSeq	5'-GTCTGGGTGCCCTCGTAG-3'
Mp64-qPCR-F	5'-CGTGCAGAACAAAGGGAAAT-3'
Mp64-qPCR-R	5'-TTCTCTTTCCGCTTCACGTT-3'
Mp64-int-F	5'-GGCTGAGAGGGAGAAGGAAC-3'
Mp64-int-Rev	5'-TTTCTTCTTCGCGTTTTCGT-3'
AtPEX4-qPCR-F	5'-AGAATGCTTGGAGTCCTGCT-3'
AtPEX4- qPCR-R	5'-TGAACCCTCTCACATCACCA-3'
Sumo_Vigs_Phusion_Frag3_F	5'-AAGGTTACCGAATTCTCTAGACAGCAAACCGGAAGACCAG-3'
Sumo_Vigs_Phusion_Frag3_R	5'-GGGACATGCCCGGGCCTCGAGTTCAGCATCACCTGCTGGTA-3'
eGFP_Fw	5'-TACCGAATTCTCTAGATGACCCTGAAGTTCATCTGC-3'
eGFP_Rv	5'-ATGCCCGGGCCTCGAGGAAGAAGTCGTGCTGCTTC-3'
NbSIZ1-qPCR-F	5'-CACCAATTCCGCCTACAACT-3'
NbSIZ1-qPCR-R	5'-GTCCCATTGTCACCCAGAAG-3'
NbEF1α-qPCR-F	5'-AGCTTTACCTCCCAAGTCATC-3'
NbEF1α-qPCR-R	5'-AGAACGCCTGTCAATCTTGG-3'
NbPP2A-qPCR-F	5'-GACCCTGATGTTGATGTTCGCT-3'
NbPP2A-qPCR-R	5'-GAGGGATTTGAAGAGAGATTTC-3'

Table S2. Plasmid constructs used in this study.

Construct	Vector	Reference	Vector specific primers for cloning	Assays used
LexA-Mp64	pLexA-N	Dualsystems DUALhybrid kit	pLexA-N-F and pLexA-N-R	Yeast two hybrid
LexA-CRN83_152/ CRN83_152_6D10	pLexA-N	Dualsystems DUALhybrid kit	pLexA-N-F and pLexA-N-R	Yeast two hybrid
Untagged Mp64	pB7WG2	Karimi et al., 2002	p35s-F and T35s-R	Arabidopsis transgenic line generation; *Phytophthora capsici* infection assay over transient overexpression in *Nicotiana benthamiana*
Untagged CRN83_152	pB7WG2	Karimi et al., 2002	p35s-F and T35s-R	*Phytophthora capsici* infection assay over transient overexpression in *Nicotiana benthamiana*
GFP-Mp64	pB7WGF2 (N-terminal GFP tag)	Karimi et al., 2002	p35s-F and GFP-Nter-F	CO-IP; Stability assay; SUMOylation assay; Subcellular localisation;
GFP-CRN83_152/ CRN83_152_6D10	pB7WGF2 (N-terminal GFP tag)	Karimi et al., 2002	p35s-F and GFP-Nter-F	CO-IP; Stability assay; SUMOylation assay; Subcellular localisation
GAD-AtSIZ1	pGAD-HA	Dualsystems DUALhybrid kit	pGAD-HA-F2 and pGAD-HA-R2	Yeast two hybrid
GAD-NbSIZ1	pGAD-HA	Dualsystems DUALhybrid kit	pGAD-HA-F2 and pGAD-HA-R2	Yeast two hybrid
AtSIZ1-RFP	pK7RWG2 (C-terminal mRFP tag)	Karimi et al., 2002	p35s-F and RFP-RevSeq	Cell death assay; Subcellular localisation
NbSIZ1-RFP	pK7RWG2 (C-terminal mRFP tag)	Karimi et al., 2002	p35s-F and RFP-RevSeq	Cell death assay; Subcellular localisation
RFP-SUMO1	pK7WGR2 (N-terminal mRFP tag)	Karimi et al., 2002	p35s-F	SUMOylation assay
AtSIZ1-myc	pGWB20 (C-terminal 10xMyc tag)	Nakagawa et al., 2007	pGWB-F and attB2	CO-IP; Stability assay; SUMOylation assay
NbSIZ1-myc	pGWB20 (C-terminal 10xMyc tag)	Nakagawa et al., 2007	pGWB-F and attB2	CO-IP; Stability assay; SUMOylation assay

Table S3. Candidate targets of effectors Mp64 and CRN83_152 based on Yeast-two-Hybrid screening.

Effector	Protein candidate Targets	Hits	Pfam domains
Mp64	ref|XP_009617749.1|PREDICTED: E3 SUMO-protein ligase SIZ1 isoform X3 [*Nicotiana tomentosiformis*]	2	SAP domain (IPR003034)<br>Zinc finger, RING/FYVE/PHD-type (IPR013083)<br>Zinc finger, FYVE/PHD-type (IPR011011)<br>Zinc finger, PHD-type (IPR001965)<br>Zinc finger, PHD-finger (IPR019787)<br>Zinc finger, MIZ-type (IPR004181)<br>Peptidase A2A, retrovirus, catalytic (IPR001995)
	ref|XP_009773140.1|PREDICTED: protein LIGHT-DEPENDENT SHORT HYPOCOTYLS 10-like [*Nicotiana sylvestris*]	2	ALOG domain (IPR006936)
	ref|XP_009773624.1|PREDICTED: uncharacterized protein LOC104223818 [*Nicotiana sylvestris*]	3	NAD(P)-binding domain (IPR016040)<br>NmrA-like domain (IPR008030)<br>Galactose-binding domain-like (IPR008979)<br>NADH:ubiquinone oxidoreductase intermediate-associated protein 30 (IPR013857)
	ref|XP_009786006.1|PREDICTED: protein LIGHT-DEPENDENT SHORT HYPOCOTYLS 10-like [*Nicotiana sylvestris*]	3	ALOG domain (IPR006936)
	ref|XP_009600988.1PREDICTED: 60S ribosomal protein L35-like [*Nicotiana tomentosiformis*]	2	none
	ref|XP_009790934.1PREDICTED: chlorophyll a-b binding protein 40, chloroplastic-like [*Nicotiana sylvestris*]	1	Chlorophyll a/b binding protein domain (IPR023329)
	ref|XP_009780849.1|chloroplast stem-loop binding protein of 41 kDa b, chloroplastic [*Nicotiana sylvestris*]	1	NAD(P)-binding domain (IPR016040)<br>NAD-dependent epimerase/dehydratase, N-terminal domain (IPR001509)
	ref|XP_009777441.1|PREDICTED: magnesium-protoporphyrin IX monomethyl ester [oxidative] cyclase, chloroplastic [*Nicotiana sylvestris*]	1	Domain<br>Ferritin-like superfamily (IPR009078)<br>Rubrerythrin (IPR003251)
	ref|XP_009776620.1|serine/threonine-protein phosphatase 2A 65 kDa regulatory subunit A beta isoform-like [*Nicotiana sylvestris*]	2	Armadillo-type fold (IPR016024)<br>Armadillo-like helical (IPR011989)
CRN83_152	E3 SUMO-protein ligase SIZ1 (Niben101Scf04549g09015.1)	3	zf-MIZ (pfam02891); SAP (pfam02037); PHD (pfam00628)

	Structure-specific endonuclease subunit SLX1 (Niben101Scf01771g01024.1)	2	GIY-YIG (pfam01541)
	Dynein light chain 2 (Niben101Scf06556g00002.1)	2	Dynein_light (pfam01221)
	Zinc finger protein (Niben101Scf02509g06003.1)	2	zf-H2C2_2 (pfam13465)
	4-hydroxy-3-methylbut-2-enyl diphosphate reductase (Niben101Scf33689g00006.1)	2	LytB (pfam02401)
	Inactive poly [ADP-ribose] polymerase (Niben101Scf02375g00013.1)	1	RST (pfam12174)
	Uncharacterised protein (jgi|Phyca11|103964|e_gw1.8.750.1)	1	
	14-3-3-like protein GF14 (Niben101Scf00477g00017.1)	1	14-3-3 (pfam00244)
	General regulatory factor 12 (Niben101Scf09559g02008.1)	1	14-3-3 (pfam00244)

References:

Karimi M, Inzé D, Depicker A. 2002. GATEWAY vectors for *Agrobacterium*-mediated plant transformation. *Trends in Plant Science* **7**(5): 193-195.

Nakagawa T, Suzuki T, Murata S, Nakamura S, Hino T, Maeo K, Tabata R, Kawai T, Tanaka K, Niwa Y, et al. 2007. Improved Gateway binary vectors: high-performance vectors for creation of fusion constructs in transgenic analysis of plants. *Biosci Biotechnol Biochem* **71**(8): 2095-2100.

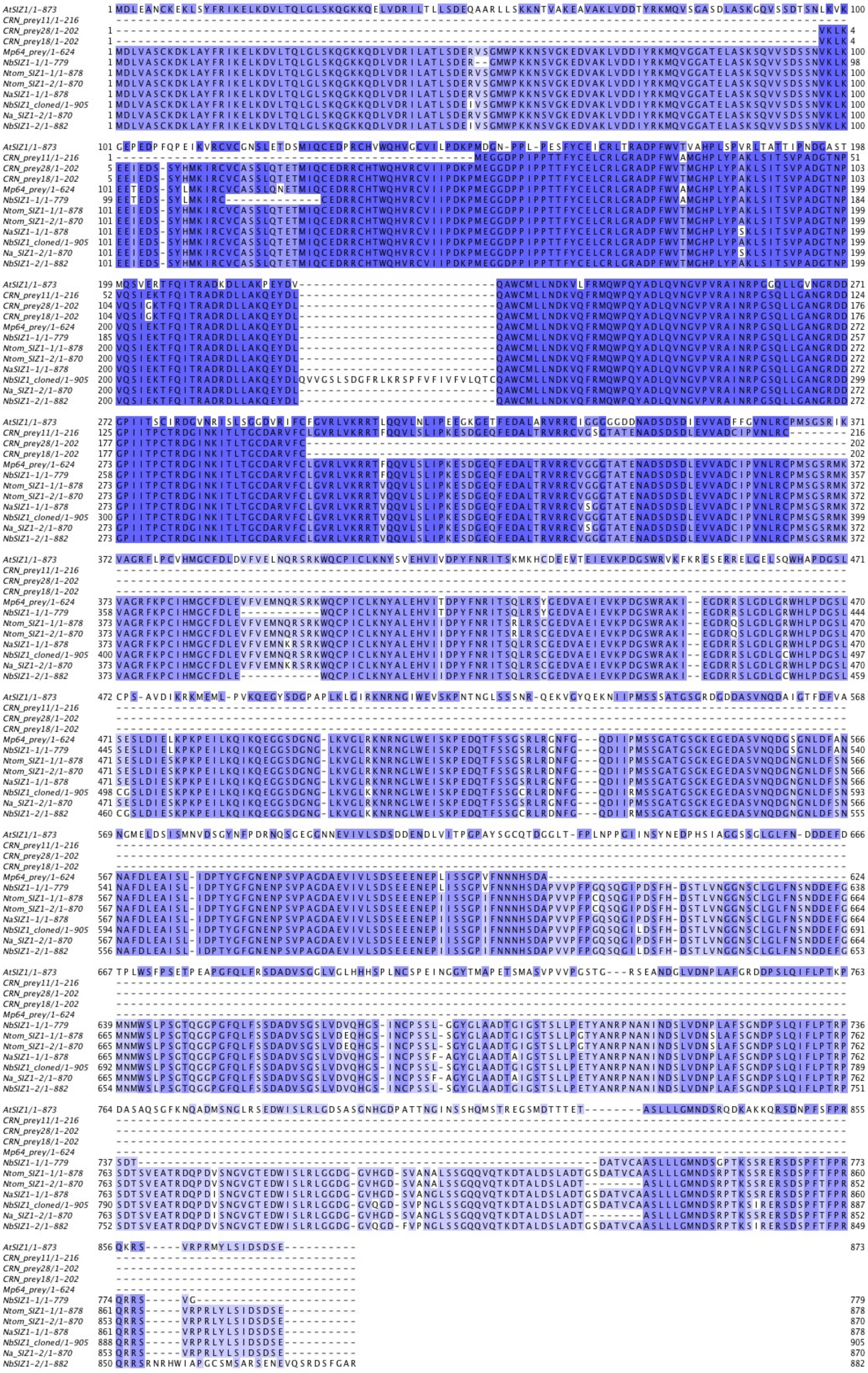
AtSIZ1/1-873
CRN_prey11/1-216
CRN_prey28/1-202
CRN_prey18/1-202
Mp64_prey/1-624
NbSIZ1-1/1-779
Ntom_SIZ1-1/1-878
Ntom_SIZ1-2/1-870
NaSIZ1-1/1-878
NbSIZ1_cloned/1-905
Na_SIZ1-2/1-870
NbSIZ1-2/1-882

Fig. S1. Amino acid alignment of different versions of SIZ1 and fragments recovered from yeast-two-hybrid screens. Three different prey sequences were recovered for yeast clones from the CRN83_152 screen (11, 18 and 28) and two identical prey sequences were recovered from yeast clones for the Mp64 screen. The CRN83_152 prey clones were only sequenced at the 5'-end. NbSIZ1-cloned corresponds to the NbSIZ1 sequence cloned and used in this study. AtSIZ1 corresponds to AT5G60410.1, NbSIZ1-1 corresponds to Niben101Scf15836g01010.1, NbSIZ1-2 corresponds to Niben101Scf04549g09015.1, Ntom_SIZ1-1 corresponds to XP_018631065.1, Ntom_SIZ1-2 corresponds to XP_018631066.1, NaSIZ1-1 corresponds to XP_019237907.1, NaSIZ1-2 corresponds to XP_019237903.1. Dark blue colour indicates high similarity, light blue colour low similarity.

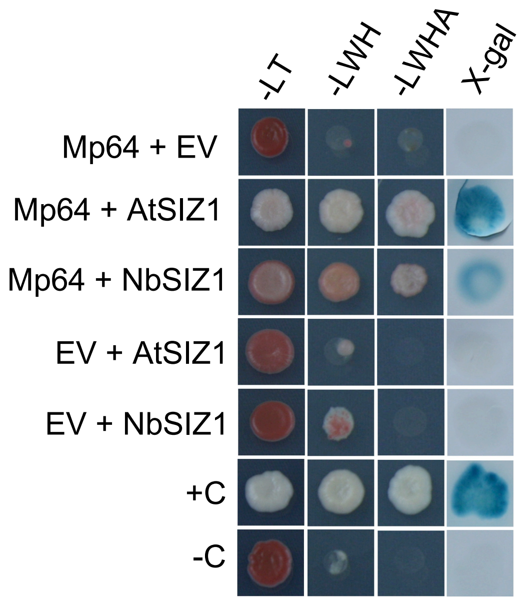

Fig. S2. Confirmation of interactions between aphid effector Mp64 and AtSIZ1 (Arabidopsis thaliana) or NbSIZ1 (*Nicotiana benthamiana*) in yeast through activation of various reporter genes. Yeast co-transformants were selected on –LW media lacking leucine and tryptophan. -LWH represents selective medium lacking leucine, tryptophan and histidine (-LWH) and -LWHA represents selective medium lacking leucine, tryptophan, histidine and adenine. X-gal assays confirmed activation of the *lacZ* reporter gene. +C and -C indicate the positive and negative controls respectively for reporter activation, and EV indicates bait or prey vector with no insert.

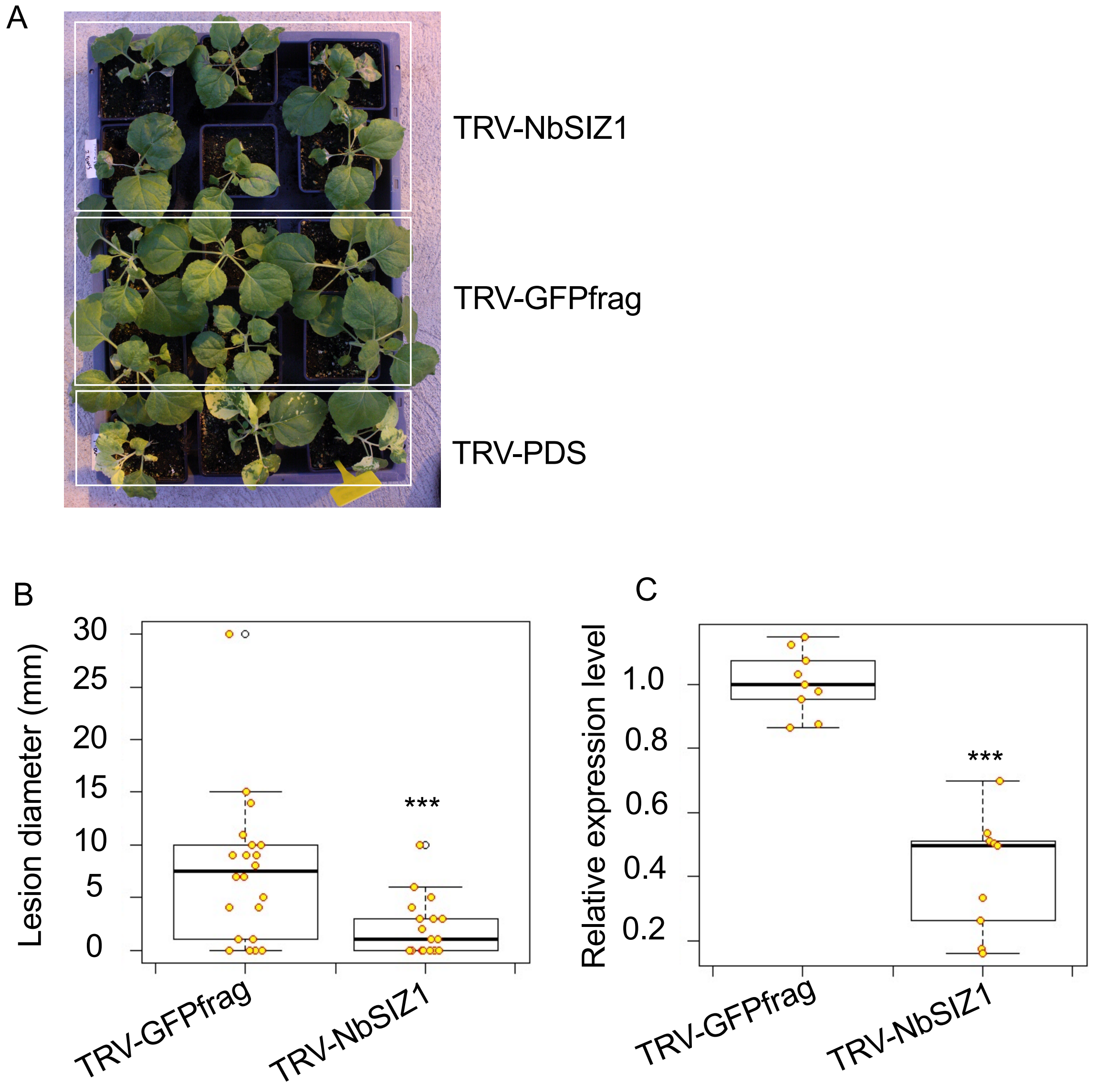

Fig. S3. Virus-induced gene silencing of *NbSIZ1* is effective and reduces host susceptibility to *Phytophthora capsici*.

(A) Representative images showing the phenotype of plants expressing TRV-*NbSIZ1* and TRV-*GFP-frag*. *NbSIZ1*-silenced plants (TRV-*NbSIZ1*) shows a slightly reduced growth and increased cell death associated virus infection compared to the control (TRV-*GFPfrag*). TRV-P*DS* was infiltrated alongside as a positive control.

(B), Lesion diameter was significantly reduced in plants expressing TRV-*NbSIZ1* compared to TRV-*GFPfrag* control. Two biological replicates (n=12 per replicate) were combined in the dataset. *N. benthamiana* leaves were inoculated with 10µl of a *P. capsici* zoospores suspension (100,000 spores/ml). The lesion diameter was recorded four days post inoculation. Asterisks denote significant difference between TRV-*NbSIZ1* and TRV-*GFPfrag* (Mann-Whitney U test, $p<0.001$).

(C) Boxplot showing that the relative expression level of *NbSIZ1* is significantly reduced in *NbSIZ1*-silenced plants (TRV-*NbSIZ1*) compared to control plants (TRV-*GFPfrag*). RT-qPCR was conducted with three biological replicates. Orange circles are individual datapoints. The black line within the box represents the median. The top and bottom edges of the box indicate

upper quantile and lower quantile. The datapoints out of the upper and lower extreme of the whisker are outliners. Expression level of *NbSIZ1* was normalized against the expression of reference genes *NbPP2A* and *NbEF1α* using ΔΔCt analysis. Asterisks denote significant difference between treatments and control (Mann-Whitney U test, p<0.001).

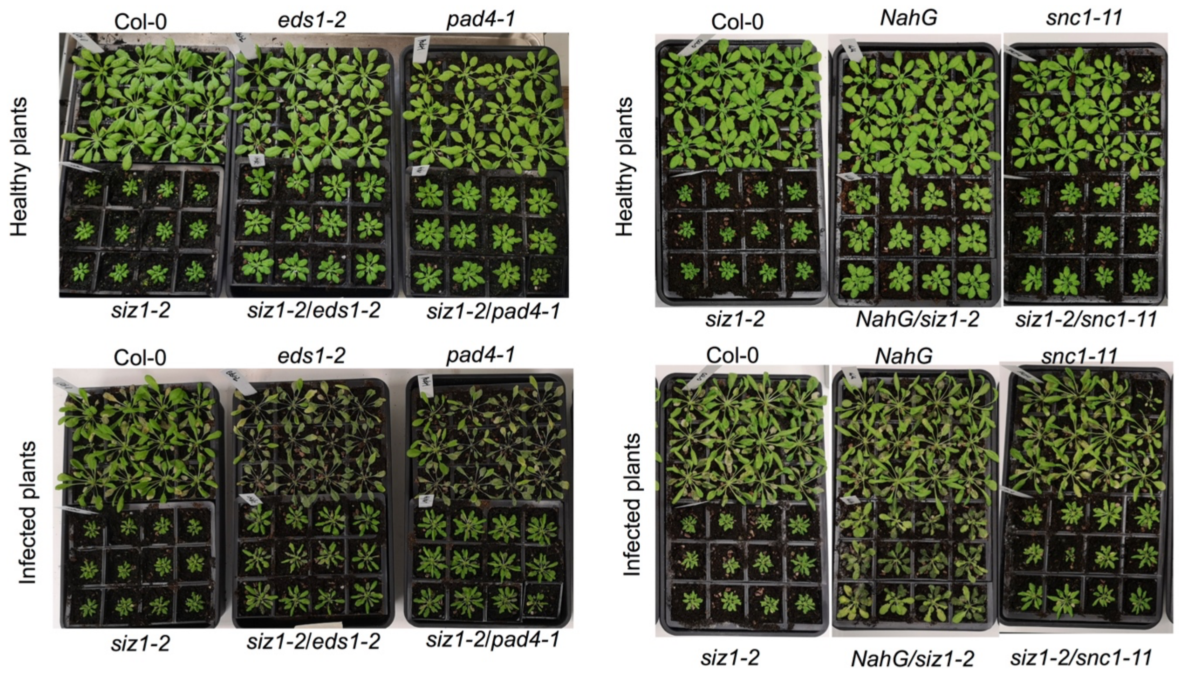

Fig. S4. Representative images of Arabidopsis mutant lines infected with *Phytophthora capsici*. The plants were spray-inoculated with zoospore suspension of 100,000 spores/ml and the images were taken 8 days post infection.

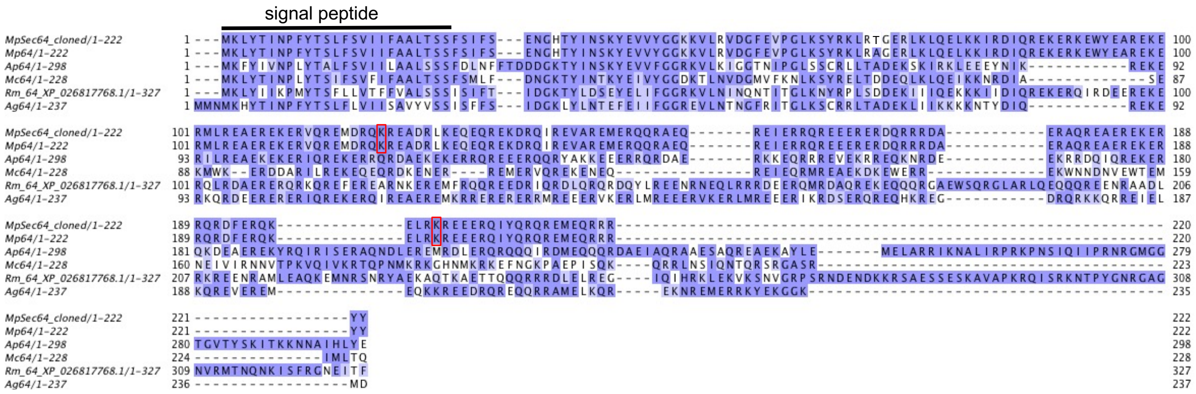

Fig. S5. Amino acid alignment of Mp64 and predicted orthologs in aphid species. Mp64-cloned indicates the cloned effector sequence used in this study. Mp64 indicated the *Myzus persicae* database sequences XP_022180025.1. Ap64 indicates the *Acyrthosiphon pisum* sequence XP_008179242.1. Rm64 indicates the *Rhopalosiphum maidis* sequence XP_026817768.1, and Ag64 indicates the *Aphis glycines* sequence KAE9543816.1. Dark blue colour indicates high similarity, light blue colour low similarity. Signal peptide sequence of Mp64 is indicated by a black line. Red boxes indicate predicted SUMOylation sites based on GPS-SUMO 2.0 predictions with medium threshold setting.

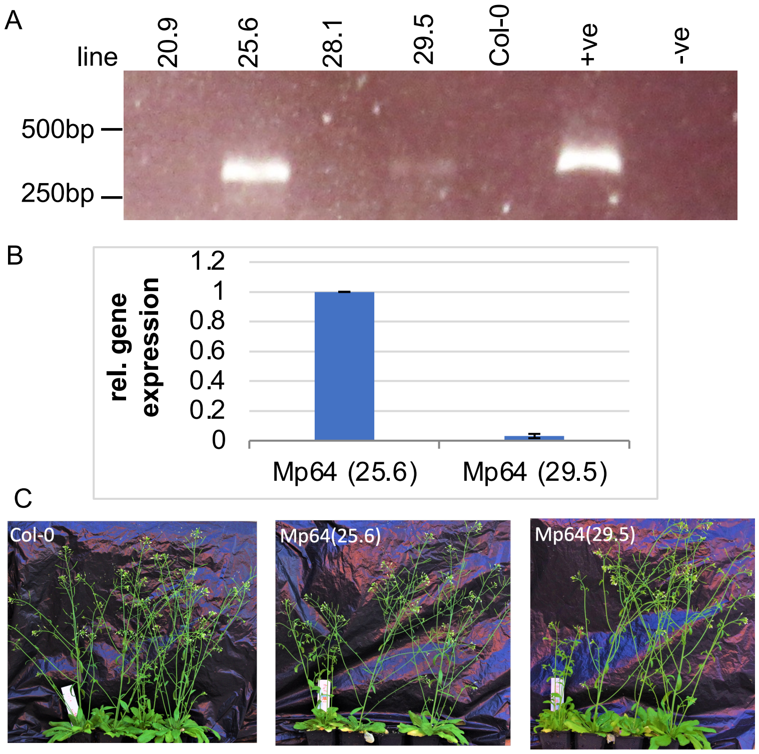

Fig. S6. Reverse transcriptase-Polymerase Chain Reaction (RT-PCR) confirms expression of aphid effector Mp64 in transgenic Arabidopsis lines and plant phenotypes.

(A) cDNA from four independent *Arabidopsis thaliana* overexpressing Mp64 lines (20.9, 25.6, 28.11, and 29.5) was used as a template for PCR with primers specific to Mp64 alongside with cDNA from Col-0, plasmid DNA with an Mp64 insert (positive control) and DNAse/RNAse treated water (negative control). The samples were analysed on a 1% agarose gel. The products from the cDNA template and plasmid were detected at the correct size of 274 bp.

(B) Relative expression level of Mp64 in two independent *Arabidopsis thaliana* overexpressing line 25.6 and 29.5. RT-qPCR was conducted with three technical replicates. Error bars indicate standard error. Expression level of Mp64 was normalised against the expression of a reference gene *AtPEX4* using ΔΔCt analysis.

(C) Representative images showing the phenotype of two selected Arabidopsis Mp64 transgenic lines (line 25.6 and line 29.5).

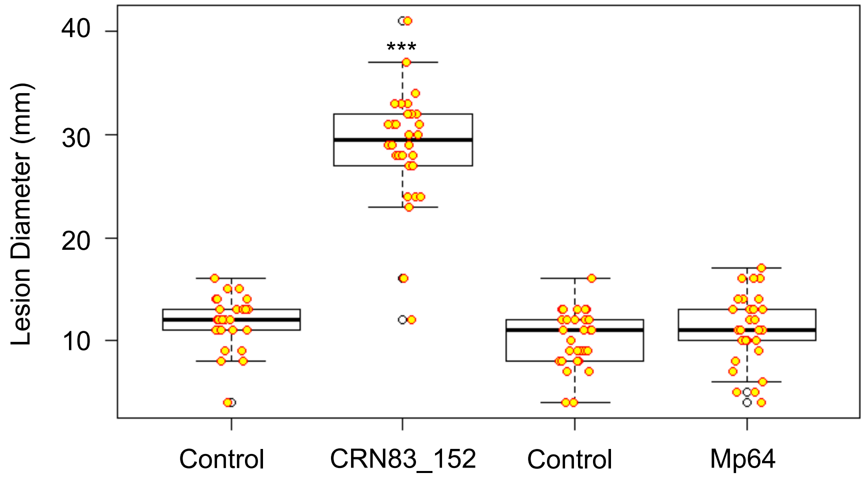

Fig. S7. Expression of CRN83_152 but not Mp64 in *Nicotiana benthamiana* enhances host susceptibility to *Phytophthora capsici*. Leaves expressing CRN83_152, Mp64 and vector controls upon agroinfiltration were challenged with 5μl *P. capsici* zoospore suspension (50,000 spores/mL) and the lesion diameter was measured two days post inoculation. A total of 30 infiltration sites per treatment were analyzed (n=30). Asterisks denote significant difference of lesion diameter between the CRN83_152 overexpressing leaves and vector control (Mann-Whitney U test, p<0.001).

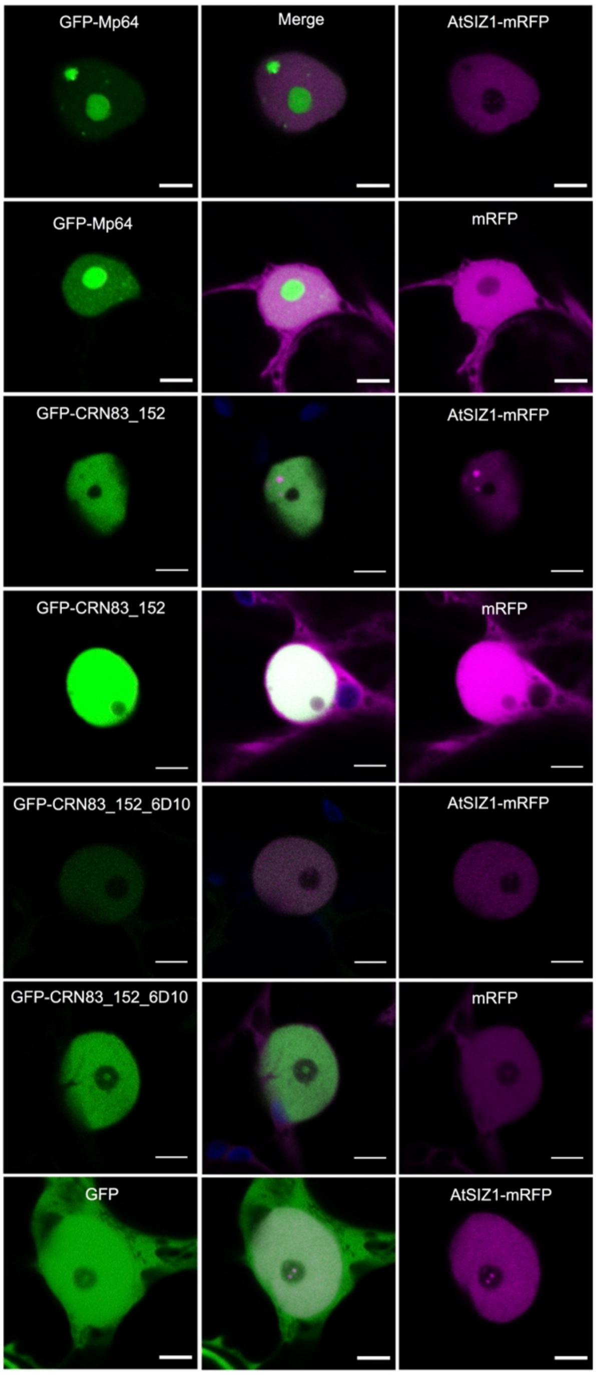

Fig. S8. Localisation of effectors Mp64 and CRN83_152(6D10) and AtSIZ1 in the host nucleus.

Leaves transiently expressing GFP-Mp64, GFP-CRN83_152 or GFP-CRN83_152_6D10 in combination with RFP or AtSIZ1 –RFP were used for confocal imaging around 36 hours after agroinfiltration. Images show single optical sections through nuclei co-expressing the GFP-effector with AtSIZ1-RFP or RFP control. Scale bars represent 5 µm.

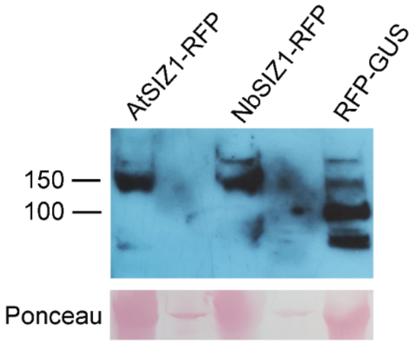

Fig. S9. Expression of full length SIZ1-RFP fusion proteins *in planta*.

Western blots showing SIZ1-RFP proteins are expressed as full-length fusion proteins. Leaf samples from infiltration sites in *N. benthamiana* were collected 3 days after infiltration. Leaf samples were ground in sample buffer and equal amounts were loaded on an SDS-PAGE gel for western blotting with an RFP-antibody. Marker indicated molecular weight in kD. Ponceau staining shows equal loading and transfer.

Replicate 2

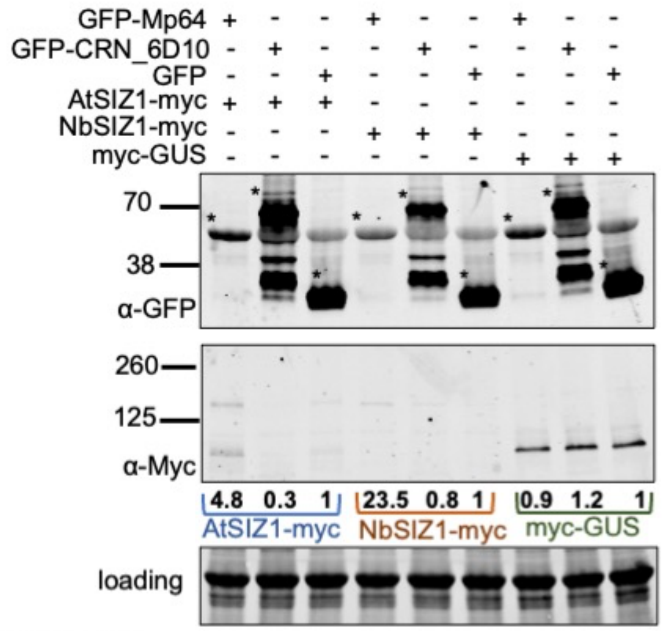

Replicate 3

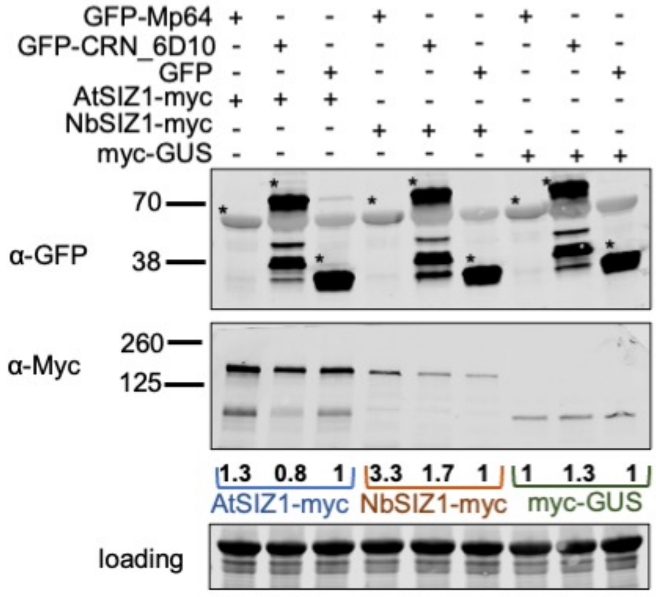

Fig. S10. Mp64 but not CRN83_152_6D10 enhances SIZ1 stability *in planta.*
Additional replicates of western blots shown in Fig. 6.
Western blots showing Mp64 increases SIZ1 protein levels. Blots were prepared using total plant extracts of *N. benthamiana* infiltration sites expression GFP-Mp64/CRN83-152_D610 or GFP (control) with SIZ1-myc. Leaf material was harvested 2 dpi. Total protein amount was detected using the Revert™ 700 Total Protein Stain followed by imaging in the 700 nm channel using an Odyssey® CLx Imaging System. The panel indicated by "loading" shows a proportion of the membrane that includes Rubisco. Detection of GFP-fusion and SIZ1-myc fusion proteins upon antibody incubation was in the 800nm channel using an Odyssey® CLx Imaging System. Asterisk indicated bands corresponding to GFP-effectors/GFP. SIZ1 protein quantitation was done by normalizing the band intensity of SIZ1-myc to the total protein amounts using Empiria Studio 2.1 (Licor). SIZ1-myc levels in samples with GFP-Mp64 or GFP-CRN83_152_6D10 were compared to GFP (control, set at 1) to generate band intensity ratios, indicated by values below the western blot incubated with Myc-antibodies.

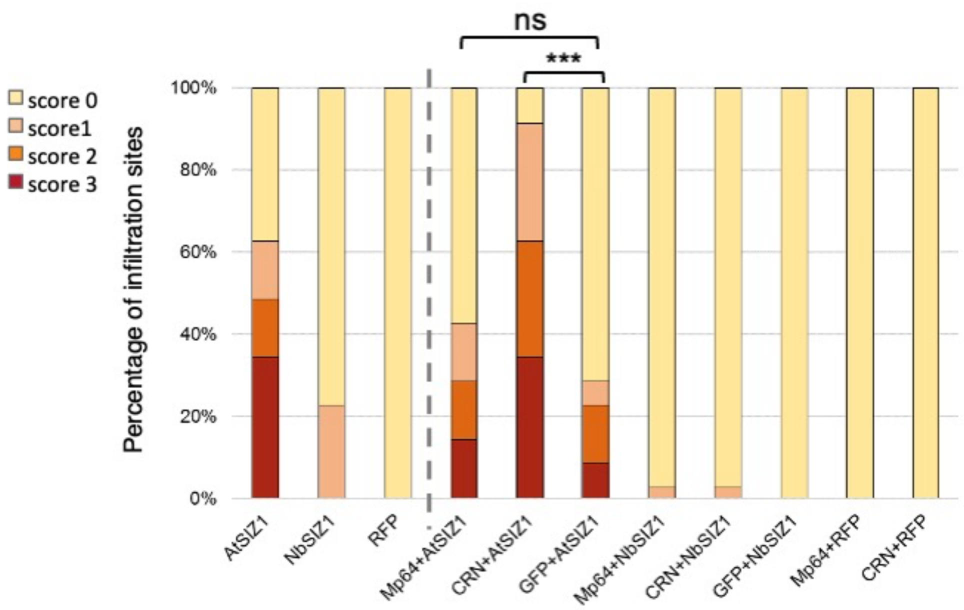

Fig. S11. CRN83_152_6D10 enhances AtSIZ1 triggered cell death in *Nicotiana benthamiana*.

Bar graph showing the proportions of infiltration sites with different levels of cell death upon expression of SIZ1 (with C-terminal RFP tag) either alone or in combination with aphid effector Mp64 or *Phytophthora capsici* effector CRN83_152_6D10 (both effectors with GFP tag), or a GFP control. Data was collected 4 days after infiltration. Graph represents data from a combination of 2 biological replicates of 11-12 infiltration sites per experiment (n=23). Note that the third biological replicate, not included here, did not yet show cell death symptoms at 4pdi. *** indicates p<0.001 (Kruskal-Wallis test with post-hoc Dunn's test for multiple comparison,) and ns indicates no significant difference.

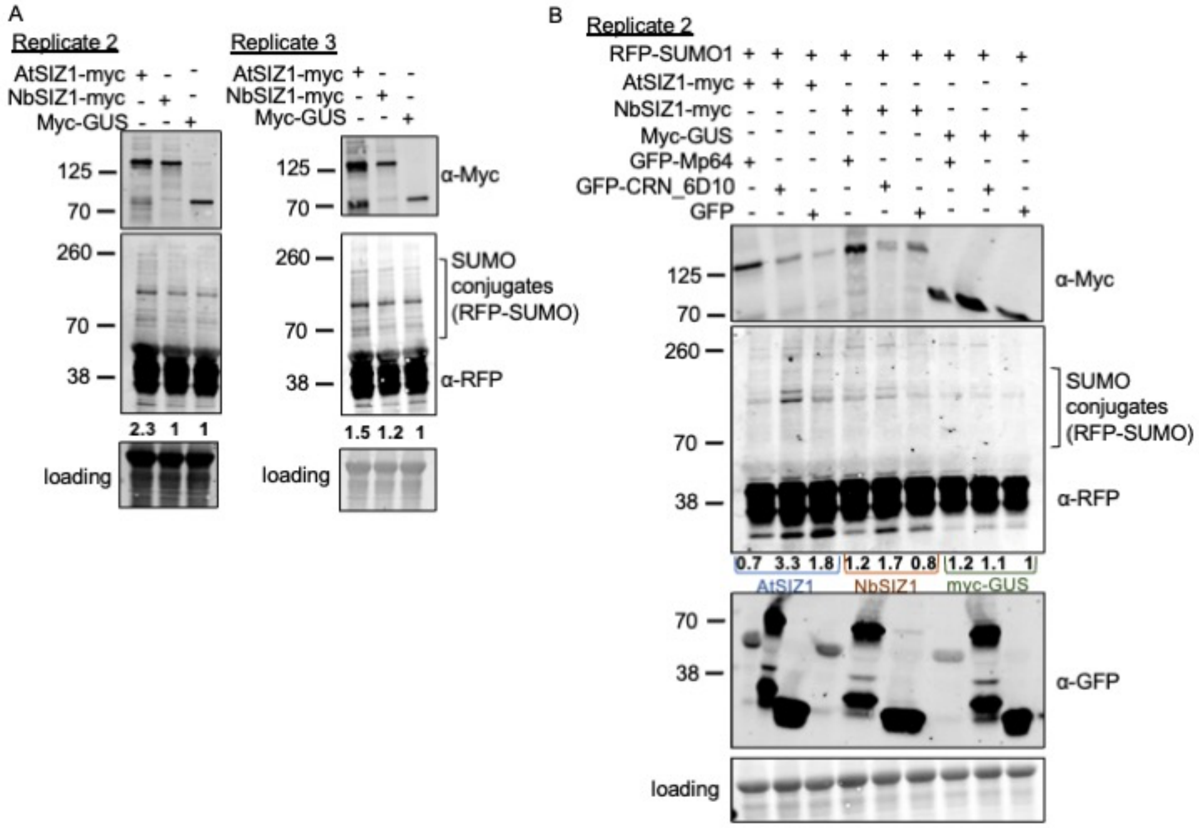

Fig. S12. CRN83_152_6D10 enhances SIZ1-mediated SUMOylation

(A) Western blot showing levels of SUMO-conjugates, detected using an RFP-antibody against RFP-AtSUMO1, upon ectopic/overexpression of AtSIZ1-myc, NbSIZ1-myc or myc-GUS (control). Blots were prepared using total plant extracts of *N. benthamiana* infiltration sites expression different SIZ1-myc versions or GUS-myc (control). Leaf material was harvested 2 dpi after 1 hour of heat stress in a 37C incubator. Total protein amount was detected using the Revert™ 700 Total Protein Stain followed by imaging in the 700 nm channel using an Odyssey® CLx Imaging System. The panel indicated by "loading" shows a proportion of the membrane that includes Rubisco. Detection of SIZ1-myc fusion proteins as well as RFP-AtSUMO1 upon antibody incubation was in the 800nm channel using an Odyssey® CLx Imaging System. Quantification of SUMO-conjugates was done by normalizing the total band intensity of the area indicated to correspond to RFP-SUMO-conjugates to the total protein amounts using Empiria Studio 2.1 (Licor). RFP-SUMO1-conjugate levels in samples with SIZ1-myc were compared to myc-GUS(control, set at 1) to generate band intensity ratios, indicated by values below the western blot incubated with RFP-antibodies.

(B) Western blot showing levels of SUMO conjugates as in (A) in the presence of GFP-Mp64, GFP-CRN83_152_6D10 or GFP (control). Western blotting and protein detection as in (A) was used to detect the presence of SIZ1-myc and GFP-effector proteins and compare levels of RFP-SUMO1-conjugates. RFP-SUMO1-conjugate levels in samples with GFP-Mp64 or GFP-CRN83_152_6D10 were compared to GFP combined with myc-GUS (control, set at 1) to generate band intensity ratios, indicated by values below the western blot incubated with RFP-antibodies.
